# Effect of IGF1 on Myogenic Proliferation and Differentiation of Bovine Skeletal Muscle Satellite Cells Through PI3K/AKT Signaling Pathway

**DOI:** 10.3390/genes15121494

**Published:** 2024-11-21

**Authors:** Xin Li, Yang Cao, Yu Liu, Wenwen Fang, Cheng Xiao, Yang Cao, Yumin Zhao

**Affiliations:** 1 Institute of Animal Husbandry and Veterinary Medicine, Ji Lin Academy of Agricultural Sciences, Gongzhuling 136100, China; lixin-hd@163.com (X.L.); cyang0508@163.com (Y.C.); liuy@cjaas.com (Y.L.); allen_wwfang@163.com (W.F.); xiaocheng@cjaas.com (C.X.); 2 Institute of Animal Biotechnology, Ji Lin Academy of Agricultural Sciences, Gongzhuling 136100, China

**Keywords:** cultivated meat, IGF1, bovine skeletal muscle satellite cells, myogenic differentiation, overexpression lentivirus, siRNA, PI3K/AKT

## Abstract

**Background**: Cultivated meat, an alternative to conventional meat, has substantial potential for alleviating environmental and ethical concerns. This method of manufacturing meat involves the isolation of skeletal muscle satellite cells (SMSCs) from donor animals, after which they proliferate in vitro and differentiate into primitive muscle fibers. The aim of this research was to evaluate how the insulin-like growth factor 1 (IGF1) gene regulates the myogenic differentiation of bovine skeletal muscle satellite cells (bSMSCs). **Methods**: bSMSCs isolated from newborn calves were cultured to the third generation in vitro and differentiated into myoblasts via the serum withdrawal method. An overexpression lentivirus and siRNA targeting the IGF1 gene were constructed and transduced into bSMSCs, which were subsequently analyzed via real-time fluorescence quantitative PCR(qRT–PCR) and Western blots. The mRNA and protein levels of the myogenic differentiation markers myosin heavy chain (MyHC) and myogenin (MyoG) were determined. **Results**: The results revealed that the lentivirus overexpressing the IGF1 gene significantly increased the expression of MyHC and MyoG, whereas the expression of both the MyHC and MyoG mRNAs and proteins was strongly reduced by si-IGF1. **Conclusions**: IGF1 positively regulates the myogenic differentiation of bSMSCs. This study provides a reference for further elucidating the molecular mechanism by which the IGF1 gene regulates the myogenic differentiation of bSMSCs via the PI3K/Akt signaling pathway and lays a foundation for establishing a regulatory network of bovine muscle growth and development.

## 1. Introduction

Cultivated meat is a new field of research that has resulted in major alterations to the current traditional meat production system. This method exploits the sustainable development of animal cells in vitro and is superior to traditional meat production in terms of water usage, the environment, land use and ethics. In theory, four types of cells can regenerate muscle tissue in vitro: muscle satellite cells (SCs) [1], embryonic stem cells (ESCs) [2], mesenchymal stem cells (MSCs) [3] and induced pluripotent stem cells (iPSCs) [4].

Muscle tissue has strong regenerative ability and plasticity, and these biological properties are dependent mainly on the occurrence of muscle formation by skeletal muscle satellite cells (SMSCs) [5,6]. Loss of skeletal muscle function, especially abnormal muscle differentiation, can result in a range of diseases, including cancer and diabetes [7]. In domesticated animals, such as cattle, SMSCs are also closely related to meat production and muscle quality [7]. SMSCs are myogenic stem cells that exist between the muscle membrane and basal membrane and show self-renewal and differentiation [8]. Generally, SMSCs are in a mostly resting state, but after injury to adult muscle tissue, SMSCs are activated and thus can undergo myogenic differentiation and can form new muscle fibers to replace injured muscle tissue [9,10]. Myogenesis is regulated by many factors, such as myogenic regulatory factors (MRFs), the myocyte enhancer factor 2 (MEF2) gene family, insulin-like growth factor (IGFs), and the serum response factor (SRF). At present, several signaling pathways, such as the Wnt, PI3K-Akt and MAPK signaling pathways, are known to play important regulatory roles in mammalian skeletal muscle development.

The IGF family, as a family of endocrine hormones and autocrine/paracrine growth factors, can regulate the development of tissues and organs, especially in the skeletal muscle metabolism, and growth synthesis and catabolism play important roles. Insulin-like growth factor 1 (IGF1) is a key growth factor that regulates the synthetic and catabolic pathways of skeletal muscle, especially carbohydrate and lipid metabolism, to support the metabolic needs of the muscle [11,12]. IGF can promote cells in the G0 and G1 phases to cross the R point and enter the M phase, thus repairing the injured site [13], indicating that IGF plays a very important role in promoting cell proliferation and differentiation in skeletal muscle injury repair. IGF1 promotes protein production in skeletal muscle and regulates cell survival, differentiation and growth in muscle tissue by activating the PI3K/Akt/mTOR and PI3K/Akt/GSK3β signaling pathways [14]. Additionally, PI3K/Akt suppresses the transcription of FoxOs and E3 ubiquitin ligases, which control the ubiquitin proteasome system (UPS)-mediated degradation of proteins.

In this study, the expression of IGF1 in bovine skeletal muscle satellite cells (bSMSCs) at the early stage (D0), middle stage (D3) and late stage (D7) of myogenic differentiation was determined via qRT–PCR and Western blotting. The overexpression lentivirus and siRNA of IGF1 were constructed, and the expression levels of the IGF1 gene, myosin heavy chain (MyHC) and myogenin (MyoG), marker gene myogenic differentiation, and the PI3K/AKT signaling pathway-related proteins PI3K, p-PI3K, AKT and p-AKT were evaluated. We explored the regulatory mechanism of IGF1 in the myogenic differentiation of bSMSCs via the PI3K/AKT signaling pathway to provide a reference for establishing the regulatory network of bovine muscle growth and development and providing a basis for the molecular breeding of bovine muscle traits.

## 2. Materials and Methods

### 2.1. Ethics Statement

The Laboratory Animal Management and Experimental Animal Ethics Committee of the Jilin Academy of Agricultural Sciences (AWEC2023A01, 9 March 2023) approved all procedures involving the animals.

### 2.2. Test Materials

The bSMSCs used in the experiment were derived from newborn calves and stored in the Laboratory of Cell Biology, Institute of Animal and Veterinary Medicine, Jilin Academy of Agricultural Sciences.

### 2.3. Reagents and Media

Trypsin (Gibco, New York, NY, USA), collagenase IV (Sigma, St. Louis, MO, USA), fetal bovine serum (FBS), horse serum (HS) (two from Gibco, New York, NY, USA), Dulbecco’s Modified Eagle Media: Nutrient Mixture F-12 (DMEM/F12), penicillin–streptomycin, Phosphate Buffer Saline (PBS) (three from HyClone, Logan, UT, USA), reverse transcriptase (TaKaRa, Dalian, China), goat serum for sealing (Beijing Zhongshan Jinqiao Company, Beijing, China), anti-MHC (Developmental Studies Hybridoma Bank, Washington, DC, USA), and FITC-labelled goat anti-mouse secondary antibodies (Boorson Company, Beijing, China) were used.

Basic medium: DMEM/F12 + 10% FBS + 1% penicillin–streptomycin.

Myogenic differentiation medium: DMEM/F12 + 2% HS.

### 2.4. In Vitro Culture and Myogenic Differentiation of bSMSCs

Previously published methods [6] were used to isolate, purify, culture and identify bSMSCs in vitro. After being resuscitated, bSMSCs were inoculated in T25 cell bottles and transmitted to the 3rd generation. When the percentage of confluent cells reached 60–70%, the basic medium was replaced with myogenic differentiation medium. For the next seven days, the induction process was continuous, with the differentiation medium being added every two days.

The expression of myogenic differentiation marker MyHC was detected by immunofluorescence assay. bSMSCs of 70% fusion were solidified with 4% paraformaldehyde at room temperature for 15 min and washed with PBS 3 times, permeated with 0. 25% Triton X-100 for 15 min, washed 3 times with PBS again, then sealer closed with a 10% goat serum for 1 h. Incubation was conducted with murine-derived monoclonal MyHC (1:100) at 4° Covernight. After washing with PBS 3 times, FITC-labeled goat was resistant to mouse secondary antibody (1:100) incubated at room temperature for 1 h. bSMSCs were washed 3 times with PBS and incubated at room temperature with DAPI. The differentiation and myotube formation of bSMSCs were observed under confocal microscope after PBS cleaning for 15 min.

### 2.5. Construction of the IGF1 Gene siRNA

Based on the mRNA sequence of the bovine IGF1 gene (NM_001077828.1), three RNAi (small RNA interference) target sites, 18, 122 and 240, were selected to design primers and then synthesized, and the sequences of the primers are shown in Table 1. These primers were named IGF1-BOS-18, IGF1-BOS-122 and IGF1-BOS-240 and were synthesized by Shanghai Gima Pharmaceutical Technology Co., Ltd., Shanghai, China, with a meaningless codon sequence used as a negative control.

### 2.6. Lentiviral Packaging and Titer Determination

The CDS region amplified fragment of the IGF1 mRNA sequence (NM_001077828.1) was cloned into the overexpression vector pLV-EF1a-EGFP(2a) Puro. The overexpression vector and control vector pLV-EF1a-EGFP-PGK-Puro were transfected into 293T cells together with the lentivirus packaging plasmid. The target virus pLV-EF1a-IGF1-P2A-EGFP(2a) Puro and the control virus pLV-EF1a-EGFP-PGK-Puro were generated, and viral titers were determined via qPCR.

Virus-infected 293T cells. After the genomic DNA was extracted, qPCR was used to quantify the number of copies of the characteristic single-copy gene X of the virus and the characteristic single-copy gene Y of the cell. The average number of viral copies in each cell was calculated, multiplied by the number of cells at the moment of infection and divided by the volume of virus to obtain the titer of the sample to be measured. The titers (infection titers) of the control and target viruses were 1.19 × 10^8^ TU/mL and 1.2 × 10^8^ TU/mL, respectively.

### 2.7. Cell Transfection

The bSMSCs were inoculated into six-well plates, three plates of which were transfected with overexpression lentivirus and the other three with siRNA. The bSMSCs were supplemented with the overexpression or control lentivirus suspension at a ratio of 1:100 and then cultured in an incubator at 37 °C and 5% CO_2_ after being shaken evenly. After culture for 24 h, the virus-containing culture medium was removed, and the basic culture medium was replaced for 48 h. Puromycin was added to the cells, which were cultured for 24 h. Then, the medium was replaced, and the dead cells without infection were removed. A total of 5 × 10^5^ cells were inoculated into a six-well plate, and the siRNA which could effectively silence the IGF1 gene of the three siRNAs mentioned in 2.5 was transfected with Lip3000. Six hours later, the basic culture medium was replaced, and the cells were cultured at 37 °C in a 5% CO_2_ incubator. After attachment of the cells to the wall, the culture medium was replaced with myogenic differentiation medium supplemented with 2% horse serum for seven days. During this time, the morphology of the cells was examined under a microscope.

### 2.8. Real-Time Fluorescence Quantitative PCR, qRT–PCR

The mRNA expression of the IGF1 gene was measured via qRT–PCR before and after myogenic differentiation, along with the MyHC and MyoG markers. Total RNA was extracted from preinduction (D0) and postinduction (D7) cells, with 3 replicates per group, and reverse-transcribed into cDNA. The reverse transcription product was diluted 5 times with ddH_2_O and used for qRT–PCR. The primers were synthesized by Shanghai Shenggong Bioengineering Company, and the sequences are shown in Table 2. The PCR system was as follows: SYBRTaq, 10 μL; cDNA, 2 μL; upstream and downstream primers, 0.4 μL; ddH_2_O supplementation to a total volume of 20 μL. The reaction procedure was 95 °C for 2 min, 95 °C for 3 s, 60 °C for 30 s, for a total of 40 cycles; 95 °C for 15 s, 65 °C for 1 min, and 95 °C for 15 s.

### 2.9. Western Blot

The protein samples were separated by 12% SDS–PAGE and transferred to PVDF membranes after the cells were lysed via RIPA lysis buffer, and the protein content was determined via the BCA technique. The primary antibodies diluted with TBST were as follows: IGF1, MyHC, MyoG, and GAPDH. The samples were incubated with the antibodies at 4 °C overnight and then incubated with secondary anti-rabbit IgG HRP-linked antibody or anti-mouse IgG HRP-linked antibody at room temperature for 1 h, after which the proteins were visualized with an enhanced chemiluminescence (ECL) hypersensitive solution.

### 2.10. Statistical Analysis

All the data are expressed as the standard deviation ± mean. In all the statistical studies, significance was set at *p* < 0.05. For every statistically significant result, GraphPad Prism 7 (GraphPad Prism software, Inc., La Jolla, CA, USA) was used to conduct one-way ANOVA and post hoc testing. Each mean was derived from three separate studies.

## 3. Results

### 3.1. Myogenic Differentiation and Immunofluorescence Identification of bSMSCs

**Figure 1:**
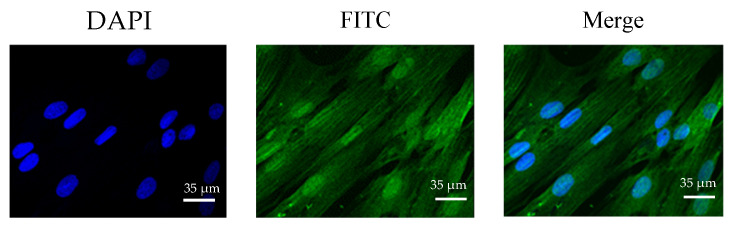
Immunofluorescence of MyHC in bSMSCs on the 7th day (600×).

After 7 days of myogenic induction of bSMSCs, the formed muscle tubes were observed under a microscope. Immunofluorescence analysis of the myogenic differentiation marker MyHC revealed positive expression, indicating that bSMSCs could fuse into muscle tubes (Figure 1).

### 3.2. Analysis of IGF1 Gene Expression During Myogenic Differentiation

**Figure 2:**
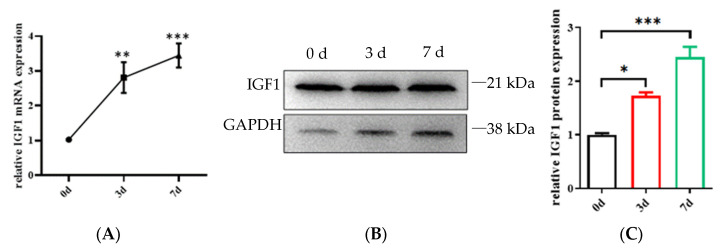
Detection of IGF1 expression in the different stages of myogenic differentiation. (**A**) The mRNA expression of IGF1 on the days of 0, 3th and 7th. (**B**,**C**) The protein expression of IGF1 on the days of 0, 3th and 7th. * *p* < 0.05, ** *p* < 0.01, *** *p* < 0.001.

The IGF1 mRNA and protein expression levels were determined via qRT–PCR and Western blotting at days 0, 3 and 7 of myogenic differentiation. The findings demonstrated that the expression level of the IGF1 gene gradually increased during myogenic differentiation, and the expression level was highest on the seventh day (Figure 2A,B).

### 3.3. siRNA Screening of the IGF1 Gene

**Figure 3:**
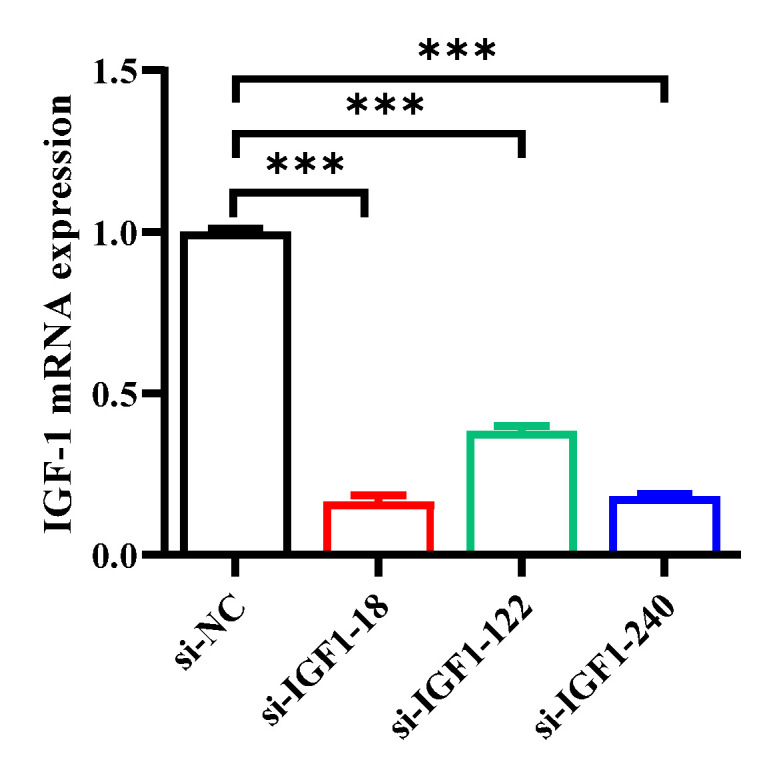
IGF1 mRNA expression levels after transfection with IGF1 siRNA. *** *p* < 0.001.

To determine the impact of the three siRNAs, we transfected these three siRNAs into bSMSCs with the negative control. After 6 h, the culture medium was changed completely, and after 12 h, qRT–PCR was used to determine the effectiveness of the transfection. The transfection effects are shown in Figure 3. Compared with that in the control group, the transfection efficacy of the siRNAs in the three groups was greater and reached more than 60%. Compared with the other two groups, the IGF1-BOS-18 group presented the lowest IGF1 mRNA expression, the best transfection effect and the best silencing effect. Therefore, IGF1-BOS-18 was selected for the follow-up test.

### 3.4. Determination of the Effect of Lentivirus Infection on IGF1 Gene Overexpression

**Figure 4:**
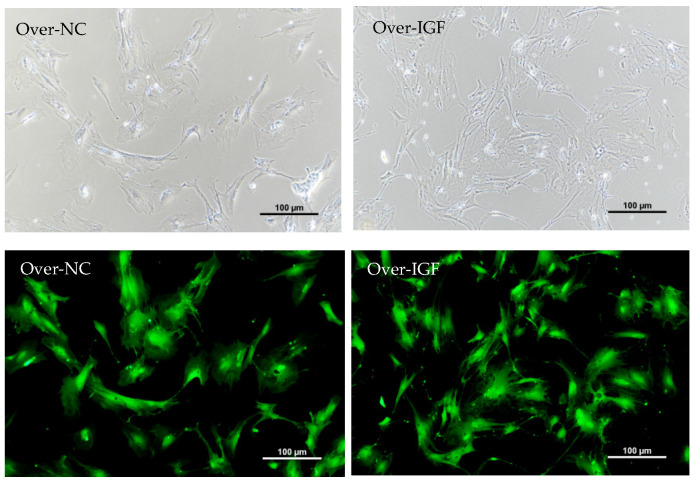
Determination of the transfection efficacy of the IGF1-overexpressing lentivirus.

After overexpression and control lentivirus infection for 24 h, green fluorescent protein expression in the cells was observed under a microscope. The results showed that after lentivirus stabilization screening, approximately 70% of the cells presented fluorescent expression, indicating that IGF1 overexpression via lentivirus infection was successful (Figure 4).

### 3.5. Effects of the IGF1 Gene on the Expression of the Proliferation Marker Gene Pax7 in bSMSCs

**Figure 5:**
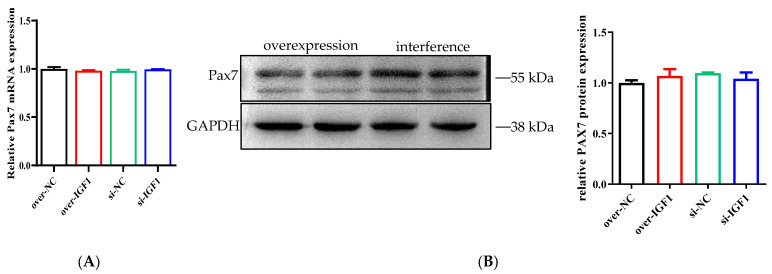
(**A**) Determination of the mRNA levels of Pax7, a marker gene of proliferation. (**B**) Determination of Pax7 protein levels, which are a marker of proliferation.

The results revealed that, after induction, IGF1 overexpression and interference had no effect on the mRNA or protein expression of paired box gene 7 (Pax7), a proliferative marker gene of bSMSCs (Figure 5A,B). The results showed that the IGF1 gene had no effect on the proliferation of bSMSCs.

### 3.6. The IGF1 Gene Positively Regulates the Myogenic Differentiation of bSMSCs

**Figure 6:**
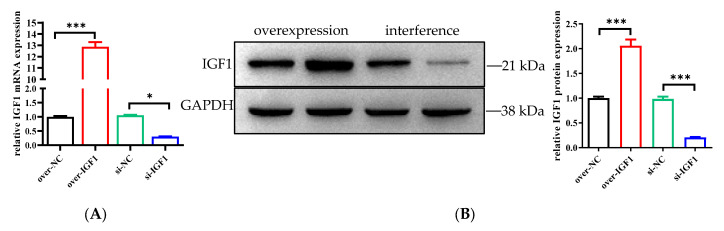
Role of IGF1 in the regulation of myogenic differentiation. (**A**) The mRNA expression of IGF1. (**B**) The protein expression of IGF1. * *p* < 0.05, *** *p* < 0.001.

After induction, the mRNA and protein expression levels in the IGF1-overexpressing group were significantly greater than those in the control group, whereas the expression levels in the IGF1 interference group were significantly lower (Figure 6A,B).

### 3.7. Effects of IGF1 Gene Overexpression and Interference on the Expression of Marker Genes Involved in the Myogenic Differentiation of bSMSCs

**Figure 7:**
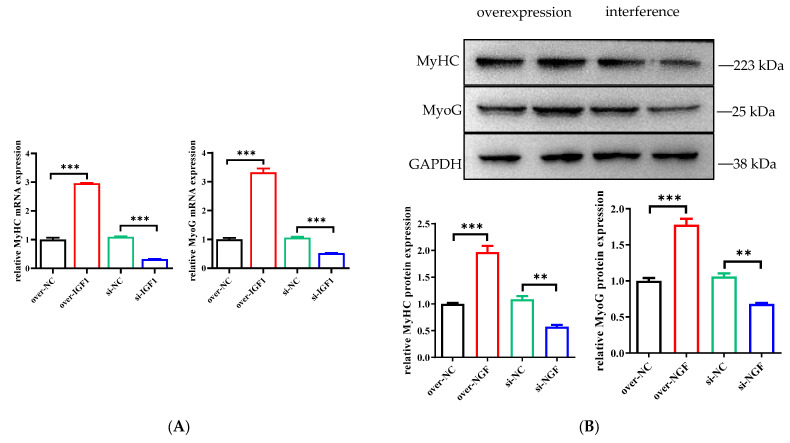
Determination of the expression levels of myogenic differentiation marker genes. (**A**) The mRNA expression of MyHC and MyoG. (**B**) The protein expression of MyHC and MyoG. ** *p* < 0.01, *** *p* < 0.001.

Following the extraction of total RNA and protein from differentiated cells, the expression levels of the myogenic differentiation marker genes MyHC and MyoG were assessed via qRT–PCR and Western blotting. Compared with those in the control group, the mRNA and protein expression levels of the myogenic differentiation markers MyHC and MyoG were much greater in the group with induced IGF1 overexpression. These findings suggest that IGF1 overexpression may have a major positive effect on these indicators. The mRNA and protein expression levels in the IGF1 interference group were drastically lower than those in the control group, and interference with the IGF1 gene significantly reduced the mRNA and protein expression of MyHC and MyoG (Figure 7A,B).

### 3.8. Effects of IGF1 Gene Overexpression and Interference on the PI3K/AKT Signaling Pathway

**Figure 8:**
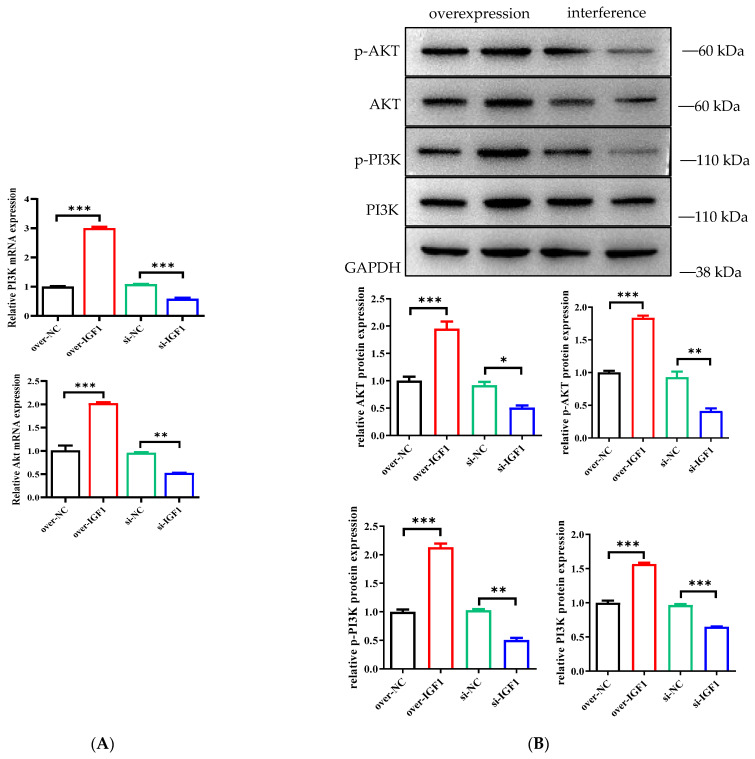
(**A**) Expression levels of genes related to the PI3k/AKT signaling pathway. (**B**) Expression levels of proteins related to the PI3k/AKT signaling pathway. * *p* < 0.05, ** *p* < 0.01, *** *p* < 0.001.

During total protein extraction, protease and phosphatase inhibitors need to be added to the protein lysate RIPA buffer to ensure that the phosphorylation signal can be efficiently detected. The Akt signaling pathway genes, proteins and their phosphorylated proteins were detected (Figure 8A,B). We found that IGF1 overexpression significantly promoted the expression of Akt (*p* < 0.001) and that siRNA-IGF1 significantly inhibited the expression of Akt (*p* < 0.001). The antibody targeting phospho-Akt at Ser473 showed that IGF1 overexpression significantly increased phosphorylation at Ser473 in the Akt pathway (*p* < 0.001), and siRNA-IGF1 significantly inhibited phosphorylation at Ser473 in the Akt pathway (*p* < 0.001).

## 4. Discussion

The main drivers of muscle regeneration, self-renewal, and hypertrophy are SMSCs, a subset of muscle-derived stem cells that are present in skeletal muscle tissue [15,16]. Various cell types exist in muscle tissue, including fibroblasts, blood cells, epithelial cells, and endothelial cells. The capacity of myogenic cells to combine to produce new muscle fibers, myogenic differentiation of local stem cells, and the production of chemokines that attract host stem cells to the injury site are major factors influencing skeletal muscle regeneration [17].

During embryonic development, many studies have shown that IGF1 plays a crucial role in muscle formation. Loss of skeletal muscle function, which is not adequately understood, is linked to heart failure, ischemia, cancer, ageing, and motor neuron degeneration. The production of IGF1 plays a key role in muscle healing and functional maintenance. Clinical trials have revealed that IGF1 is directly associated with muscle number and strength development, inhibits muscle degeneration, prevents excessive toxins from producing inflammation, and increases the proliferative capacity of SMSCs [18]. Studies have shown that myogenic differentiation of SMSCs is regulated mainly by the MAPK, AMPK and PI3K/AKT signaling pathways. AMPK negatively regulates the formation of muscle ducts, and AMPK signaling is decreased in the late stage of SMSC proliferation. The PI3K/AKT signaling pathway actually induces myogenesis through IGF1 stimulation of myogenin expression [19]. Research has demonstrated that IGF1 plays a critical role in myoblast mitotic activity, primarily by modulating two signaling pathways linked to cell cycle progression and survival: the PI3K/AKT pathway and the mitogen-activated protein kinase (MAPK/ERK1/2) pathway [20]. In addition, IGF1-mediated AKT signaling promotes protein synthesis and muscle cell development in skeletal muscle [21,22]. Both IGF-1R and AKT1/AKT2 double knockout mice presented severe growth defects and decreased amounts of skeletal muscle; however, in the IGF-1R knockout mice, these defects may have been caused by a decrease in the number of muscle cells, whereas in the Akt1/Akt2 double knockout mice, the shrinkage of individual cells was the primary cause. Moreover, another study showed that IGF-1R functions during development are dependent mainly on Akt [23]. IGF1 can be synthesized in the MSCs of rodents with muscle injury, thereby stimulating the proliferation of MSCs and myoblast differentiation [19,24].

Artificial meat is generally obtained by isolating the skeletal muscle cells of the donor animal in vitro and inducing them to proliferate and form muscle in vitro so that they develop into mature muscle tubes [25]. Numerous elements related to differentiation and proliferation control this process. Four myogenic transcription factors belonging to the MRF family—myogenic factor 5 (Myf5), myoblast determination protein 1 (MyoD1), MRF4, and MyoG—are primarily responsible for regulating myogenesis [26]. Muscle progenitor cells express MyoD to join the myogenic lineage during embryonic muscle development [27]. SMSCs also express Pax7, another myogenic regulator [28]. MyoD expression gradually increases during the activation of satellite cells to promote myocyte proliferation, whereas MyoG promotes the differentiation of satellite cells into muscle fibers [29]. MyHC is a muscle fiber motor protein and an important marker gene for the myogenic differentiation of SMSCs [30]. These growth factors function as transcription factors inside cells. Intercellular signaling, however, is also believed to promote and control the division and multiplication of cells.

According to the transcriptome sequencing results of the different stages of myoblastic differentiation of bSMSCs, our research group screened the differentially expressed gene IGF1, which was verified by qRT–PCR and was consistent with the sequencing results. The expression level of the IGF1 gene gradually increased during myogenic differentiation and was the highest on the seventh day. An IGF1-overexpressing lentivirus was constructed, and approximately 70% of the infected cells presented fluorescent expression after the lentivirus was stabilized and screened. The mRNA and protein expression levels in the induced IGF1 overexpression group were significantly greater than those in the control group, and the overexpression of the IGF1 gene significantly increased the mRNA and protein expression levels of the myogenic differentiation markers MyHC and MyoG. The expression levels of the IGF1 gene and MyHC and MyoG in the IGF1-interference group were much lower than those in the control group. After induction, IGF1 overexpression and interference had no effects on the mRNA or protein expression of the bSMSCs proliferation marker gene, Pax7. IGF1 overexpression significantly promoted Akt expression and phosphorylation at Ser473, whereas IGF1 interference significantly inhibited Akt expression and phosphorylation at Ser473.

## 5. Conclusions

The overexpression of IGF1 can increase the mRNA and protein levels of MyHC, MyoG and the genes related to the PI3K/AKT signaling pathway, whereas interference with IGF1 can decrease the mRNA and protein expression levels of MyHC, MyoG and the PI3K/AKT signaling pathway. However, the overexpression and interference of IGF1 had no effect on the proliferation of bSMSCs. In conclusion, IGF1 positively regulates the myogenic differentiation of bSMSCs. These findings lay the groundwork for further investigations of the regulatory network of the IGF1-mediated PI3K/AKT signaling pathway during the myogenic differentiation of bSMSCs.

## Figures and Tables

**Table 1:** siRNA sequences of the IGF1 gene.

siRNA	Primer Sequences (5′-3′)
Negative control	F: ATCCTCCTCGCATCTCTTCTATC
	R: TGAAATAAAAGCCCCTGTCTCC
IGF1-BOS-18	F: CCUCCUCGCAUCUCUUCUATT
	R: UAGAAGAGAUGCGAGGAGGTT
IGF1-BOS-122	F: CAGUUCGUGUGCGGAGACATT
	R: UGUCUCCGCACACGAACUGTT
IGF1-BOS-240	F: UGAGGAGGCUGGAGAUGUATT
	R: UACAUCUCCAGCCUCCUCATT

**Table 2:** Primer information for qRT–PCR.

Genes	Primer Sequence/(5′-3′)	Product Length/bp	Annealing Temperature/°C
MyHC	F: AAGCTGATGCCTTGGCTGAT	219	60
	R: TCTCTGTGGCGTGTTTCTCC		
MyoG	F: CAGTACATAGAGCGCCTGCA	235	60
	R: TCCACTGTGATGCTGTCCAC		
IGF1	F: GCTTTTGTGATTTCTTGAAGCAG	355	60
	R: TTCTTCAAATGTACTTCCTTCTGAG		
Pax7	F: ACGAAGCGGACAAGAAGGAG	211	60
	R: TCGGGTGTAGATGTCTGGGT		
PI3K	F: CTATCCTGTGCCGGCTACTG	265	60
	R: CCATGCCGGCGTAAAATCAG		
Akt	F: CATGCAGCACCGATTCTTCG	201	60
	R: CGAGTAGGAGAACTGGGGGA		
GAPDH	F: GTCGGAGTGAACGGATTCGG	238	60
	R: CCAGCATCACCCCACTTGAT		

## Data Availability

The raw data supporting the conclusions of the article will be made available by the authors without undue reservation.

## References

[1] Ben-Arye T., Levenberg S. (2019). Tissue engineering for clean meat production. Front. Sustain. Food Syst..

[2] Bogliotti Y.S., Wu J., Vilarino M., Okamura D., Soto D.A., Zhong C., Sakurai M., Sampaio R.V., Suzuki K., Izpisua Belmonte J.C. (2018). Efficient derivation of stable primed pluripotent embryonic stem cells from bovine blastocysts. Proc. Natl. Acad. Sci. USA.

[3] Beier J.P., Bitto F.F., Lange C., Klumpp D., Arkudas A., Bleiziffer O., Boos A.M., Horch R.E., Kneser U. (2011). Myogenic differentiation of mesenchymal stem cells co-cultured with primary myoblasts. Cell Biol. Int..

[4] Stanton M.M., Tzatzalos E., Donne M., Kolundzic N., Helgason I., Ilic D. (2019). Prospects for the use of induced pluripotent stem cells in animal conservation and environmental protection. Stem Cells Transl. Med..

[5] Yan J., Yang Y., Fan X., Liang G., Wang Z., Li J., Wang L., Chen Y., Adetula A.A., Tang Y. (2022). circRNAome profiling reveals circFgfr2 regulates myogenesis and muscle regeneration via a feedback loop. J. Cachexia Sarcopenia Muscle.

[6] Henrot P., Blervaque L., Dupin I., Zysman M., Esteves P., Gouzi F., Hayot M., Pomiès P., Berger P. (2023). Cellular interplay in skeletal muscle regeneration and wasting: Insights from animal models. J. Cachexia Sarcopeni Muscle.

[7] Yu H., Xing J., Zhang R. (2020). Research progress of skeletal muscle satellite cells and effects of RNAi on meat quality. Meat Ind..

[8] Yan S., Jing S., Chi S., Yan H., Chun Z., Hui L. (2012). Isolation, culture and identification of duck skeletal muscle satellite cells. Jiangsu Agric. Sci..

[9] Relaix F., Zammit P.S. (2012). Satellite cells are essential for skeletal uscle regeneration: The cell on the edge returns centre stage. Development.

[10] Li X., Yu Y., Zhang L., Ma H., Luo X., Wei T., Xiao C., Zhang Q., Cao Y., Zhao Z. (2021). Isolation, Culture, Identification and myogenic differentiation of sheep skeletal muscle satellite cells. China Anim. Husb. Vet. Med..

[11] Verhees K.J., Pansters N.A., Baarsma H.A., Remels A.H., Haegens A., de Theije C.C., Schols A.M., Gosens R., Langen R.C. (2013). Pharmacological inhibition of GSK-3 in a guinea pig model of LPS-induced pulmonary inflammation: II. Effects on skeletal muscle atrophy. Respir. Res..

[12] McPherron A.C., Lawler A.M., Lee S.J. (1997). Regulation of skeletal muscle mass in mice by a new TGF-p superfamily member. Nature.

[13] Taylor W.E., Bhasin S., Artaza J., Byhower F., Azam M., Willard D.H., Kull F.C., Gonzalez-Cadavid N. (2001). Myostatin inhibits cell proliferation and protein synthesis in C2C12 muscle cells. Am. J. Physiol. Endocrinol. Metab..

[14] Morissette M.R., Cook S.A., Buranasombati C., Rosenberg M.A., Rosenzweig A. (2009). Myostatin inhibits IGF-I-induced myotube hypertrophy through Akt. Am. J. Physiol. Cell Physiol..

[15] Stout A.J., Mirliani A.B., Rittenberg M.L., Shub M., White E.C., Yuen J.S.K., Kaplan D.L. (2022). Simple and effective serum-free medium for sustained expansion of bovine satellite cells for cell cultured meat. Commun. Biol..

[16] Derya O., Kathleen L., Cemile B., Anisha J., Krishi P., Yong M. (2024). Optimized adipogenic differentiation and delivery of bovine umbilical cord stem cells for cultivated meat. Gels.

[17] Wang Y., Song C., Yin G., Meng Y., Zhang F. (2024). Alleviation of behavioral deficits, amyloid-β deposition, and mitochondrial structure damage associated with mitophagy upregulation in AD animal models via AAV9-IGF-1 treatment. Brain Res..

[18] Song Y.H., Song J.L., Delafontaine P., Godard M.P. (2013). The therapeutic potential of IGF-I in skeletal muscle repair. Trends Endocrinol. Metab..

[19] Xu Q., Wu Z. (2000). The insulin-like growth factor-phosphatidylinositol 3-kinase-Akt signaling pathway regulates myogenin expression in normal myogenic cells but not in rhabdomyosarcoma-derived RD cells. J. Biol. Chem..

[20] Fu S., Yin L., Lin X., Lu J., Wang X. (2018). Effects of cyclic mechanical stretch on the proliferation of L6 myoblasts and its mechanisms: PI3K/Akt and MAPK signal pathways regulated by IGF-1 receptor. Int. J. Mol. Sci..

[21] Sandri M., Barberi L., Bijlsma A.Y., Blaauw B., Dyar K.A., Milan G., Mammucari C., Meskers C.G., Pallafacchina G., Paoli A. (2013). Signalling pathways regulating muscle mass in ageing skeletal muscle: The role of the IGF1-Akt-mTOR-FoxO pathway. Biogerontology.

[22] Liu M., Zhang S. (2011). Amphioxus IGF-like peptide induces mouse muscle cell development via binding to IGF receptors and activating MAPK and PI3K/Akt signaling pathways. Mol. Cell. Endocrinol..

[23] Peng X.D., Xu P.Z., Chen M.L., Hahn-Windgassen A., Skeen J., Jacobs J., Sundararajan D., Chen W.S., Crawford S.E., Coleman K.G. (2003). Dwarfism, impaired skin development, skeletal muscle atrophy delayed bone development, and impeded adipogenesis in mice lacking Akt1 and Akt2. Genes Dev..

[24] Stadhouders L.E.M., Smith J.A.B., Gabriel B.M., Verbrugge S.A.J., Hammersen T.D., Kolijn D., Vogel I.S.P., Mohamed A.D., de Wit G.M.J., Offringa C. (2023). Myotube growth is associated with cancer-like metabolic reprogramming and is limited by phosphoglycerate dehydrogenase. Exp. Cell Res..

[25] Skrivergaard S., Rasmussen M.K., Therkildsen M., Young J.F. (2021). Bovine satellite cells isolated after 2 and 5 days of tissue storage maintain the proliferative and myogenic capacity needed for cultured meat production. Int. J. Mol. Sci..

[26] Kim B., Min Y., Jeong Y., Ramani S., Lim H., Jo Y., Kim W., Choi Y., Park S. (2023). Comparison of growth performance and related gene expression of muscle and fat from LYD and Woori black pigs. J. Anim. Sci. Technol..

[27] Wardle F.C. (2019). Master control: Transcriptional regulation of mammalian Myod. J. Muscle Res. Cell Motil..

[28] Zammit P.S., Relaix F., Nagata Y., Ruiz A.P., Collins C.A., Partridge T.A., Beauchamp J.R. (2006). Pax7 and myogenic progression in skeletal muscle satellite cells. J. Cell Sci..

[29] Schmidt M., Schuler S.C., Huttner S.S., von Eyss B., von Maltzahn J. (2019). Adult stem cells at work: Regenerating skeletal muscle. Cell. Mol. Life Sci..

[30] Paula N.M., Arianna N.C., Amelia J.K., Sonu P., Michelle S.P., Jose R.P. (2024). Post-translational modifications of vertebrate striated muscle myosin heavy chains. Cytoskeleton.

